# Epidemiological characteristics of bacillary dysentery from 2009 to 2016 and its incidence prediction model based on meteorological factors

**DOI:** 10.1186/s12199-019-0829-1

**Published:** 2019-12-28

**Authors:** Qiuyu Meng, Xun Liu, Jiajia Xie, Dayong Xiao, Yi Wang, Dan Deng

**Affiliations:** 1 School of Public Health and Management, Research Center for Medicine and Social Development, Innovation Center for Social Risk Governance in Health, Chongqing Medical University, Chongqing, 400016 China; 2 Department of Healthcare-associated Infection Control, The Second Affiliated Hospital of Military Medical University, Chongqing, 400037 China; 3 Institute for Prevention and Control of Endemic and Parasitic Diseases, Chongqing Center for Disease Control and Prevention, Chongqing, 400042 China

**Keywords:** *Shigella*, Dysentery, Meteorological factors, Boruta algorithm, Genetic algorithm, Support vector regression, Predictive model, China

## Abstract

**Background:**

This study aimed to analyse the epidemiological characteristics of bacillary dysentery (BD) caused by *Shigella* in Chongqing, China, and to establish incidence prediction models based on the correlation between meteorological factors and BD, thus providing a scientific basis for the prevention and control of BD.

**Methods:**

In this study, descriptive methods were employed to investigate the epidemiological distribution of BD. The Boruta algorithm was used to estimate the correlation between meteorological factors and BD incidence. The genetic algorithm (GA) combined with support vector regression (SVR) was used to establish the prediction models for BD incidence.

**Results:**

In total, 68,855 cases of BD were included. The incidence declined from 36.312/100,000 to 23.613/100,000, with an obvious seasonal peak from May to October. Males were more predisposed to the infection than females (the ratio was 1.118:1). Children < 5 years old comprised the highest incidence (295.892/100,000) among all age categories, and pre-education children comprised the highest proportion (34,658 cases, 50.335%) among all occupational categories. Eight important meteorological factors, including the highest temperature, average temperature, average air pressure, precipitation and sunshine, were correlated with the monthly incidence of BD. The obtained mean absolute percent error (MAPE), mean squared error (MSE) and squared correlation coefficient (*R*^2^) of GA_SVR_MONTH values were 0.087, 0.101 and 0.922, respectively.

**Conclusion:**

From 2009 to 2016, BD incidence in Chongqing was still high, especially in the main urban areas and among the male and pre-education children populations. Eight meteorological factors, including temperature, air pressure, precipitation and sunshine, were the most important correlative feature sets of BD incidence. Moreover, BD incidence prediction models based on meteorological factors had better prediction accuracies. The findings in this study could provide a panorama of BD in Chongqing and offer a useful approach for predicting the incidence of infectious disease. Furthermore, this information could be used to improve current interventions and public health planning.

## Background

Bacillary dysentery (BD) is a serious infectious intestinal disease caused by *Shigella*. The disease is transmitted via the oral-faecal route or through contact with contaminated water and food. The main clinical manifestations of BD are diarrhoea, fever, abdominal colic, etc. [1, 2]. BD is one of the most common causes of diarrhoea. Christopher et al. estimated that *Shigella* infection is the second leading cause of diarrhoeal death, with approximately 164,300 deaths caused by BD worldwide in 2015. Of these deaths, 54,900 were of children under the age of 5, accounting for 12.5% of the total [3]. In China, BD imposes a considerable public health burden; nearly 123,283 cases of bacillary and amoebic dysentery occur on an annual basis, placing this disease within the top five infectious diseases in China in 2016 [4].

Clearly, the harmful effects of BD on humans cannot be ignored. We urgently need to find effective prevention and control measures. However, the risk of infectious diseases has always been affected by multiple factors, such as climate, the regional economy and environmental health. The impacts of meteorological factors on diseases, especially infectious diseases, are of particular current concern to researchers, and relevant studies have been comprehensive [5–10]. Numerous studies have concluded that BD transmission might be influenced by meteorological factors [11–14]. For example, a study in Chaoyang District, Beijing, used a structural equation model (SEM) to analyse the correlation between meteorological factors and BD incidence and showed that BD incidence was positively correlated with air temperature and negatively correlated with sunshine [11]. A study in Hunan Province, China, used a Bayesian space-time hierarchical model (BSTHM) to analyse the effect of meteorological factors on BD and found that BD incidence increased by 3.194% with every 1 °C rise in temperature and increased by 0.674% with every 1% increase in relative humidity [12]. A study in Jinan City, China, adopted a distributed lag nonlinear model (DLNM) to analyse the lagged effect of meteorological factors on BD incidence and showed that each 5 °C rise in temperature increased the number of BD cases by 61% at lag 0–lag 7 days [13]. Similarly, a study in Hefei Province, China, adopted the DLNM model to analyse the lagged effect of meteorological factors on BD. The authors found that the risk of BD increased with the temperature rise above a threshold (18.4 °C), and the temperature effects appeared to be acute; the proportion of BD attributable to hot temperatures (temperature = 31.2 °C) was 18.74% [14].

While there are many studies on the correlation between BD and meteorological factors, researchers have neglected another important issue, which is how to use the correlation between BD and meteorological factors to establish a prediction model of BD incidence. The prediction of the incidence of infectious diseases is the basis of epidemic prevention and control. It can provide decision-making support for relevant health departments to formulate solutions and reduce risk and loss, and it is of great significance in the field of disease research and the formulation of epidemic prevention and control strategies.

According to a previous study, BD incidence in some areas of Chongqing has been relatively high in recent years [15]. This suggests that it is necessary to investigate the current BD situation throughout Chongqing. Therefore, this paper consists of two parts. First, an epidemiological description of current situation of the BD epidemic in Chongqing from 2009 to 2016 is provided to demonstrate the spatial, temporal and population distribution of this epidemic. Second, based on the correlation between meteorological factors and BD incidence, prediction models for BD incidence are established, and the role of meteorological factors in improving model accuracy is discussed. The latter part is our key research content.

## Materials and methods

### Survey region

**Fig. 1:**
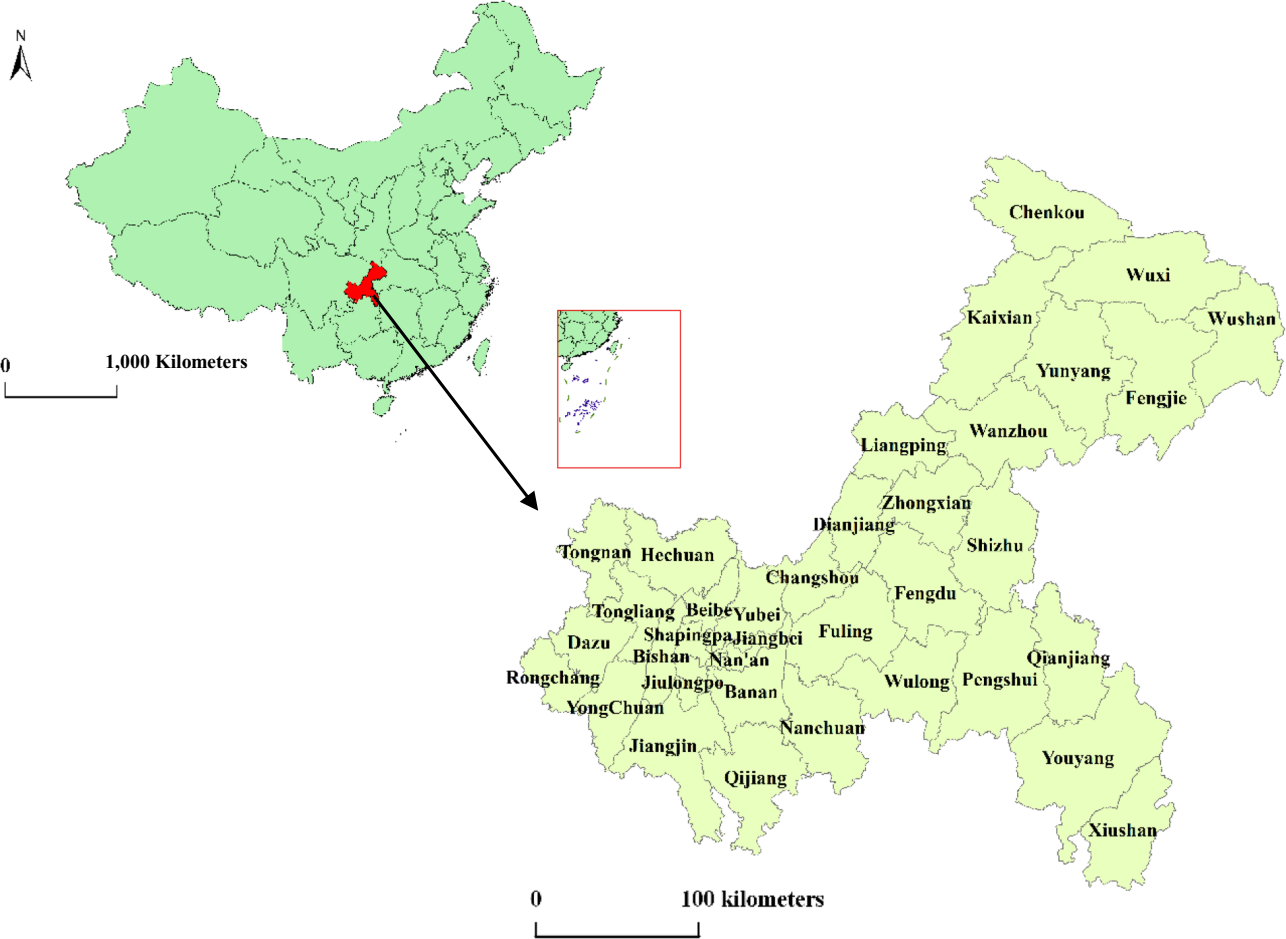
Administrative division map in Chongqing at the county level

Located in the southwestern part of inland China, Chongqing is the only municipality directly under the control of the central government in western China. Its longitude is 105° 11′~110° 11′, and its latitude is 28° 10′~32° 13′ (Fig. 1). Chongqing’s landforms are dominated by hills and mountains, which account for 76% of the area. The city has a total population of approximately 30.75 million over an area of 82,403.10 km^2^ and administers 38 counties. Chongqing has a continental subtropical monsoon humid climate, with four distinctive seasons: a cold winter, a hot summer, a spring with variable temperatures and a fall during which temperatures drop steeply. Rain is concentrated during the summer and autumn. The annual average temperature, rainfall, relative humidity and sunlight hours among the counties range from 16–18 °C, 1000–1350 mm, 70–80% and 1000–1400 h, respectively.

### Data collection

#### Bacillary dysentery cases

Daily reported data from January 1, 2009, to December 31, 2016 were obtained from Chongqing Municipal Center for Disease Control and Prevention. The data included each patient’s gender, age, occupation, address and time of BD onset. According to the diagnostic criteria for bacterial dysentery and amoebic dysentery (WS 287-2008) issued by the Ministry of Health of the People’s Republic of China, a clinically diagnosed BD case was defined based on following clinical features: fever, chills, abdominal pain, tenesmus, bloody or mucus stool, or stool containing N15/high-power field (HPF) leukocytes or purulent cells, and microscopically discernible red blood cells and phagocytic cells [16]. In this study, BD cases included clinically diagnosed cases and confirmed cases through clinical diagnosis combined with pathogenic examination. All clinical and hospital doctors must report all clinically diagnosed and confirmed BD cases to the local centres for disease control and prevention (CDC) within 24 h through the Internet-based Chinese disease control and prevention information system.

#### Meteorological data

After summarising the previous studies, we found that air temperature, relative humidity, wind speed, sunshine, relative humidity and precipitation have an impact on BD incidence [11–14, 17]. To establish a prediction model with high prediction accuracy, ten meteorological factors on a daily basis (average air temperature (°C), maximum air temperature (°C), minimum air temperature (°C), average air pressure (hPa),water vapour pressure (hPa), sunshine duration (h), relative humidity (%), precipitation (mm), 2-min wind speed (m/s) and 10-min wind speed(m/s)) were finally obtained from the Chongqing Meteorological Bureau. Chongqing Meteorological Bureau has set up 2200 general meteorological monitoring stations in the whole city and divided all general stations into 34 base stations according to their geographical location. In this study, the meteorological data of the whole city were obtained based on the average of the 34 base stations.

### Methods

#### Descriptive epidemiological analysis

Descriptive epidemiological analysis was first used to analyse the epidemiological characteristics of BD from 2009 to 2016 in Chongqing to identify the distribution of BD. The results could help researchers develop more targeted public health interventions and provide a theoretical basis for disease prevention and control.

#### Construction of predictive models

Based on the correlation between meteorological factors and BD incidence, predictive models of BD incidence were established. Before the models were established, the data of BD cases and meteorological factors were collated monthly. Eighty percent of the data sets were randomly selected as models’ training samples, while 20% of the data sets were used as models’ test samples. In this study, the incidence of dysentery was used as the dependent variable, and the number of cases and meteorological factors in the 1-month period before the predicted time point were used as the independent variables in the models. The Boruta algorithm was used for the selection of the models’ feature sets (i.e. variable sets) because the Boruta algorithm can select all of the feature sets related to the dependent variable instead of selecting a feature set that can make the model cost function the smallest for a specific model. Thus, the algorithm can help researchers understand the influencing factors of the dependent variable more comprehensively; this improves and increases the efficiency of feature selection and improves the accuracy of machine learning [18]. Then, according to the feature selection results and the hypothesis that the influence of meteorological factors on BD is complex and nonlinear, a support vector regression model (SVR) was selected to establish predictive models of BD incidence [19]. Finally, a genetic algorithm (GA) was applied to search for SVR model parameters (penalty parameter *C*, radial basis kernel function parameter *γ* and threshold *ε* of the ε-insensitive function) to improve the predictive ability of the SVR model [20].

### Statistical software

The Boruta package in R-3.5.1 was used to implement the Boruta algorithm. Based on Matlab 2016a (MathWorks), the establishment of the epidemiological analysis and the predictive model of BD were completed. Additional files 2 and 3 show the core code and data set of the establishment of predictive model. The mean absolute percent error (MAPE), mean squared error (MSE) and squared correlation coefficient (*R*^2^) were selected as the indexes used to evaluate the precision of the models. The optimal predictive model was the one in which the values of MAPE and MSE were the smallest and the value of *R*^2^ was the largest.

## Results

### Epidemiological characteristics

**Fig. 2:**
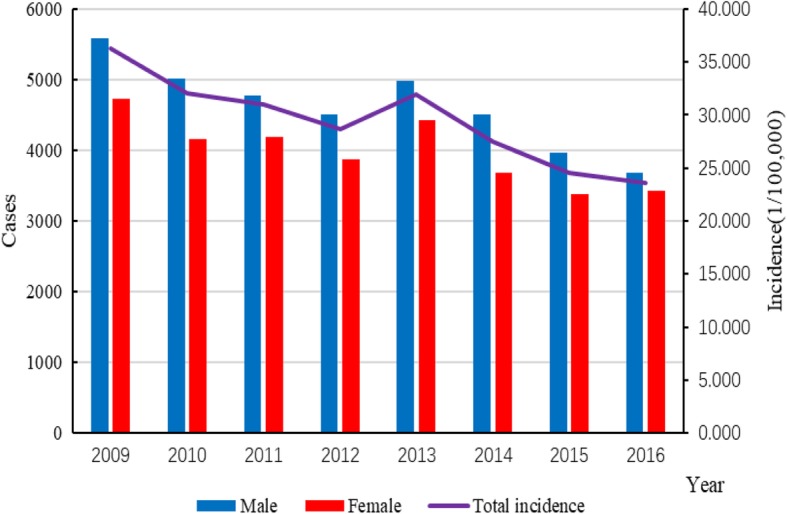
Total incidences and gender distribution of bacillary dysentery in Chongqing from 2009 to 2016 (yearly)

**Fig. 3:**
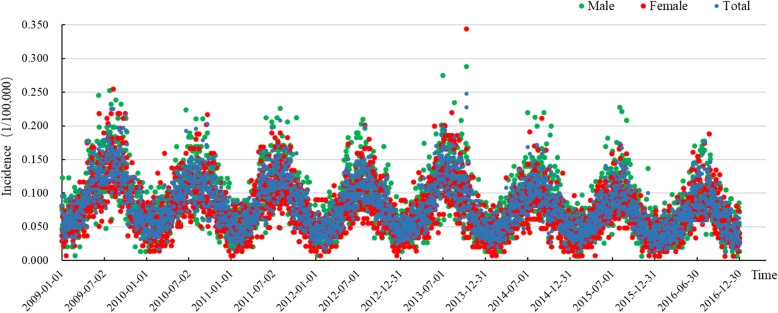
Total incidence and gender distribution of bacillary dysentery in Chongqing from 2009 to 2016 (daily)

**Fig. 4:**
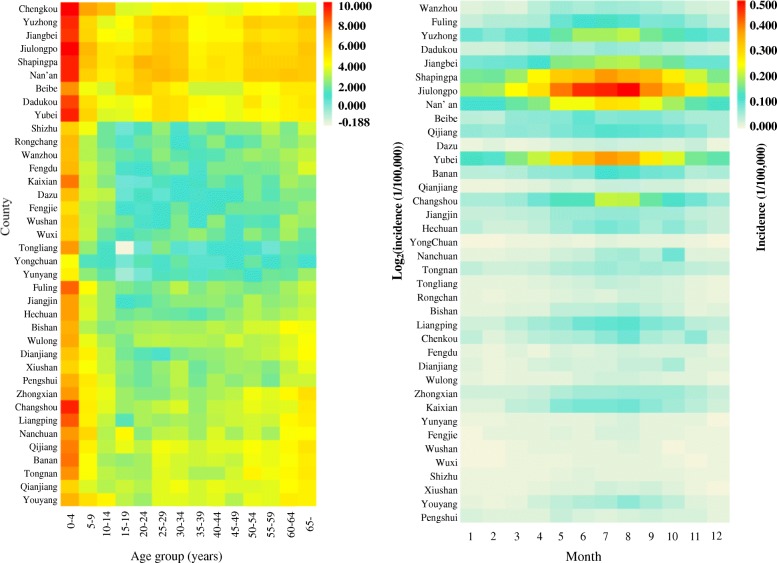
The incidence of bacillary dysentery in different age groups in Chongqing (left) and the seasonal variation in the incidence of bacillary dysentery in each region of Chongqing (right)

**Fig. 5:**
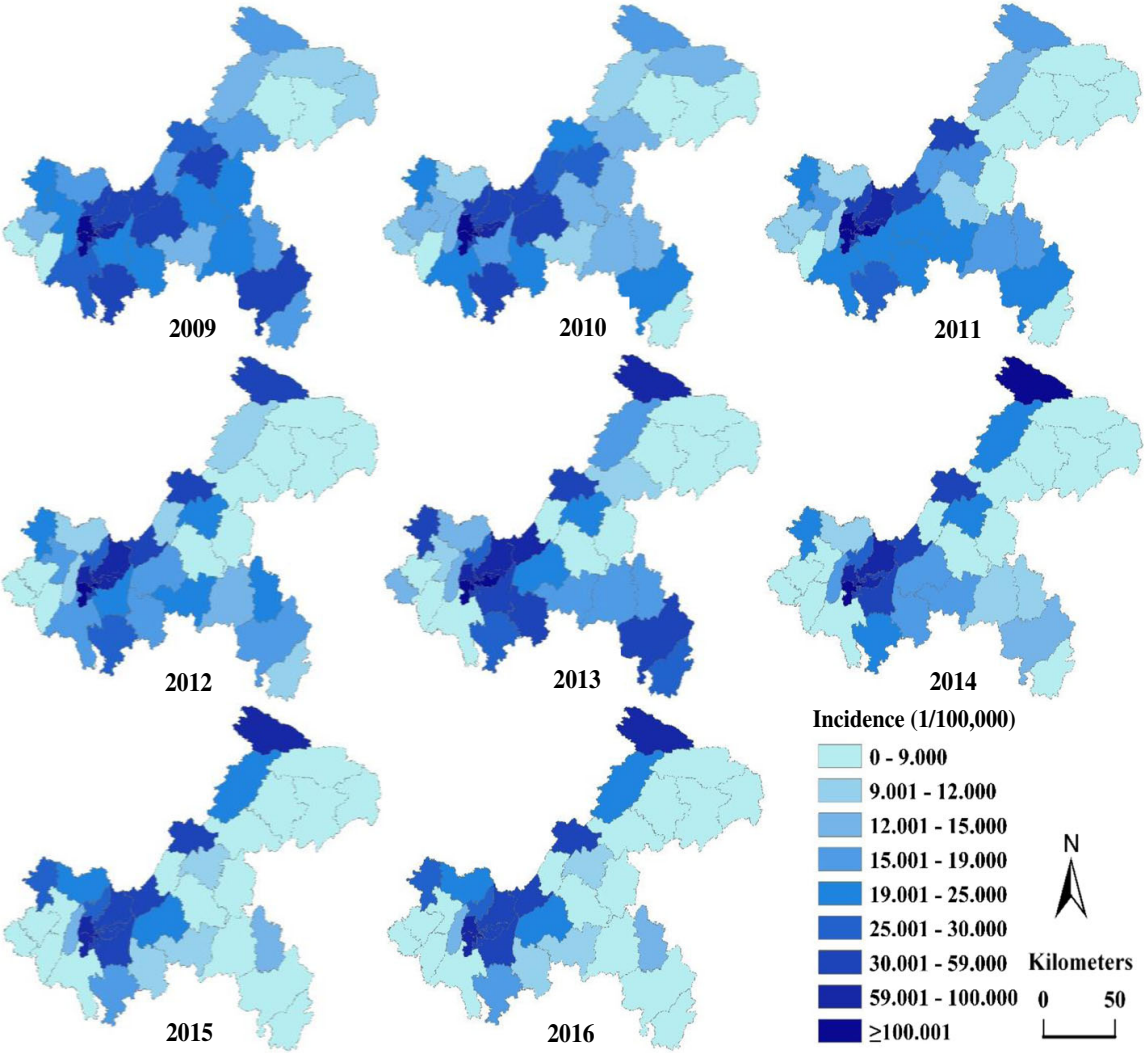
Incidence maps of bacillary dysentery in Chongqing from 2009 to 2016

The total number of reported cases of BD in Chongqing from 2009 to 2016 was 68,855; the incidence declined from 36.312/100,000 to 23.613/100,000 and the average annual incidence was 29.394/100,000 during this period (Fig. 2). The top three incidences were among population subgroups aged 0–4, 5–9 and ≥ 65 years; their incidences were 295.892/100,000; 24.938/100,000 and 22.524/100,000, respectively, which accounted for 52.879%, 4.627% and 8.925% of the total cases, respectively (Fig. 4 (left)). In the general population, the incidence in males (31.002/100,000) was higher than that in females (27.724/100,000) from 2009 to 2016, with a ratio of 1.118:1. With regard to the occupation distribution, pre-education children accounted for the most total cases (34,658 cases, 50.335%), followed by farmers (8031 cases, 11.664%) and students (5592 cases, 8.121%). In terms of the temporal distribution, BD incidence showed obvious seasonality: it peaked in the period from May to October, with 45,131 cases, accounting for 65.545% of all cases (Figs. 3 and 4 (right)). In terms of the regional distribution, the cases were mainly concentrated in the metropolitan core area and the northeastern region of Chongqing (Fig. 5).

### The prediction models for morbidity based on meteorological factors

**Fig. 6:**
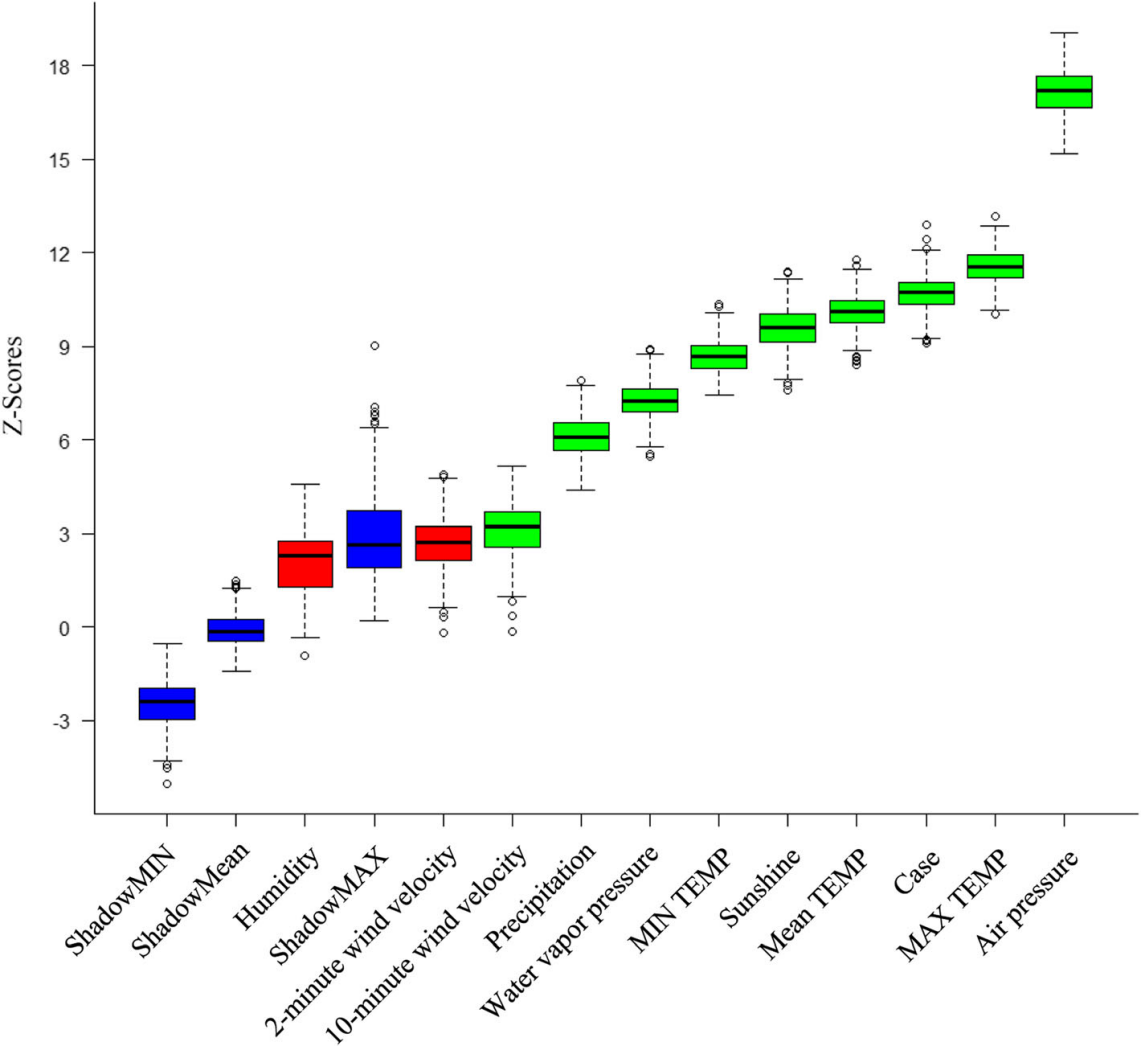
Nine variables confirmed to be important: air pressure, max TEMP (temperature), mean TEMP, MIN TEMP, sunshine, water vapour pressure, precipitation, 10-min wind velocity and case. Note: The Z-score shows the importance of variables. The shadow variable is a random variable whose value is generated by permuting the original values across observations

The result of the feature set selected by the Boruta algorithm showed that meteorological factors, except the mean relative humidity and mean 2-min wind velocity, were deemed to be related to BD incidence (Fig. 6). Therefore, we used the monthly average of 8 important meteorological factors (average air temperature, maximum air temperature, minimum air temperature, average air pressure, water vapour pressure, sunshine duration, precipitation and 10-min wind speed) and the number of BD cases as independent variables to establish the incidence prediction models.

#### Establishment and effect evaluation of monthly BD incidence prediction models

**Fig. 7:**
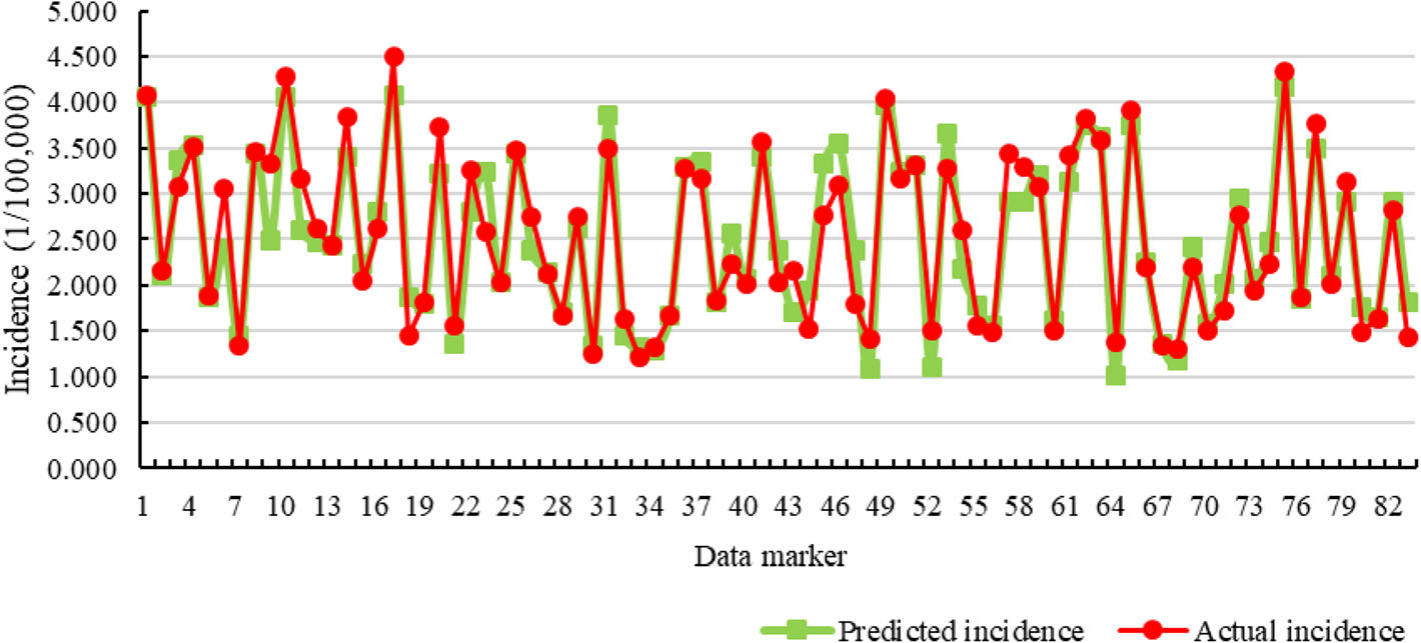
The incidence predicted by the GA_SVR_MONTH model and the actual incidence: the 10th randomly selected training and test data set

The results show that the MSE, MAPE and *R*^2^ values of the GA_SVR_MONTH model (the independent variables contained both BD incidence and meteorological factors) were 0.087, 0.101 and 0.922, respectively. For comparison with the GA_SVR_MONTH model, the GA_SVR1_MONTH model that selected only the monthly number of patients as an independent variable was established, and its MSE, MAPE and *R*^2^ were 0.214, 0.172 and 0.753, respectively. Furthermore, the SVR_MONTH model without GA optimization (the independent variables were the number of patients and meteorological factors) and the SVR1_MONTH model without GA optimization (the independent variable was only the number of patients) were established. Table 1 shows that the GA_SVR_MONTH model with the GA combined with meteorological factors was the most accurate. Compared with the GA_SVR1_MONTH, SVR_MONTH and SVR1_MONTH models, the MSE decreased by 60%, 50% and 65%, respectively; the MAPE decreased by 41%, 28% and 46%, respectively; and the *R*^2^ increased by 22%, 10% and 28%, respectively. Figure 7 shows the fitting curves of GA_SVR_MONTH on the training set and test set.

**Table 1:** Comparison of the prediction accuracy of the monthly BD incidence prediction models

Model	MSE	MAPE	*R*^2^
GA_SVR_MONTH	0.087	0.101	0.922
GA_SVR1_MONTH	0.214	0.172	0.753
SVR_MONTH	0.172	0.141	0.840
SVR1_MONTH	0.250	0.186	0.721

In this study, all models were trained 10 times (each training sample was randomly selected); thus, the values of MSE, MAPE and *R*^2^ in Table 1 are equal to the means of the corresponding predictive accuracy indicators of each of the 10 models.

## Discussion

This study found that 68,855 BD cases were reported in Chongqing from 2009 to 2016, with an average annual incidence of 29.394/100,000. Although BD incidence in Chongqing showed a downward trend overall, it was still higher than the incidence in the same period in China (20.28/100,000 in 2009, 15.29/100,000 in 2012, 11.24/100,000 in 2014) [21, 22], the USA in 2016 (6.53/100,000), Zhejiang Province between 2004 and 2015 (4.25/100,000) and Sichuan Province between 2004 and 2014 (22.12/100,000) [1, 5, 23], which highlights the serious situation regarding the prevention and control of BD in Chongqing. Chongqing has a subtropical monsoon humid climate and covers an area of 824,000 km^2^, of which 78% is mountainous; it has sufficient precipitation, a warm temperature and a suitable climate for the growth and reproduction of *Shigella*, which may be one of the reasons BD incidence in Chongqing is higher than that in other areas. From 2009 to 2016, BD incidence in the population aged 0–4, 5–9 and ≥ 65 years was relatively high in Chongqing. This is likely because the above population groups are mostly kindergarten children, primary school students, pre-education children and elderly people with weak immunity or poor hygiene habits. Furthermore, children in kindergarten institutions and schools are more concentrated; the environment is relatively closed, and it is very easy for pathogens to spread among individuals, which leads to outbreaks and relatively high prevalence rates [2, 24–26]. BD incidence was higher in men than in women in Chongqing, and the proportion of farmers was relatively large; this finding is consistent with other research results and may be attributed to that men are more likely to lack good health habits and to participate in group activities such as attending parties and dining out. Additionally, while the physical activity level of farmers is high, their working environments are frequently unsanitary [1, 5, 8, 21, 27]. This, coupled with a lack of knowledge of disease prevention and control, results in elevated incidences of BD among men and farmers. The period of high incidence of BD in Chongqing is mainly concentrated in May–October of each year. In summer and autumn, the temperature in Chongqing rises, the air pressure decreases, rainwater is abundant and flies and bacteria breed easily, which provides good conditions for the reproduction and transmission of *Shigella* [28–31]. In addition, a significant increase in temperature can not only change people’s eating habits but also have adverse effects on body temperature and metabolism. For example, people tend to eat more raw and cold food when the temperature is high, and uncooked or cold food is more likely to contain pathogens. Additionally, heatstroke, which is caused by high-temperature exposure, could affect the immune system and make people more susceptible to infectious diseases [32]. Overall, relevant health sectors should take specific steps to prevent and control BD in key groups, high-incidence periods and areas in light of the epidemiological characteristics. For example, the high-incidence districts and counties can be included in the key prevention and control plan, and the prevention and control education regarding BD should be strengthened in key groups such as children, students and farmers to prevent the spread of dysentery.

The prediction of the incidence of infectious disease plays an important role in epidemic prevention and control as it provides decision-making support to relevant health sectors, enabling those sectors to formulate solutions and reduce the risk of an epidemic [33]. Therefore, this study has important significance in the field of disease research and the formulation of epidemic prevention and control strategies. In this paper, based on the correlations between meteorological factors and BD incidence, the Boruta algorithm and GA combined with the SVR model were used to establish predictive models of BD incidence. Our study fills a gap left by previous studies that did not fully consider the correlations between meteorological factors and BD incidence.

We chose the Boruta algorithm for feature selection because it is an efficient algorithm based on random forests. It can select all relevant variables to facilitate the establishment of a high-accuracy BD incidence predictive model. As Kursa et al. indicated, finding all relevant attributes, instead of only non-redundant ones, may be very useful. This is particularly necessary when one is interested in understanding mechanisms related to the subject of interest instead of merely building a black box predictive model [18]. Numerous diseases and environmental exposure-related studies have applied this algorithm for variable or feature selection [34–38]. The Boruta algorithm has been proven to be an efficient and stable feature selection algorithm. Although the feature set selected by the Boruta algorithm may contain highly collinear independent variables, such as minimum temperature, maximum temperature and mean temperature, multicollinearity of independent variables is not a problem for constructing prediction models [39]. Moreover, the BD prediction model we constructed shows satisfactory prediction accuracy, which also indicates that the Boruta algorithm is applicable to the selection of variables in this study.

The reason why we chose the SVR model to establish BD incidence prediction models is that the SVR model has good performance in solving regression problems. Sapankevyc et al. reviewed 66 studies that applied the SVR model to explore time series prediction and concluded that SVR has good performance in time series data prediction. Especially when the time series data have the characteristics of typical non-stationary and nonlinear data, the prediction effect of the SVR model is much better than that of other mathematical statistics and nonlinear analysis methods [40]. The reason could be that SVR has the global optimal solution when solving a model equation, while other methods (such as BP neural network) cannot guarantee the global minimum error value when optimising a network. In this paper, the MSE, MAPE and *R*^2^ of the GA_SVR_MONTH model reached 0.087, 0.101 and 0.922, respectively, which indicated high predictive accuracy of the model. By comparing the models established in this paper, the importance and rationality of meteorological factors as characteristic variables of the model used to predict BD incidence were confirmed. Furthermore, we used the same methods to establish predictive models of weekly BD incidence (GA_SVR_WEEK). The MSE, MAPE and *R*^2^ of the GA_SVR_WEEK model, whose independent variables are BD cases and meteorological factors 4 weeks prior to the predictive week, reached 0.006, 0.110 and 0.888, indicating satisfactory prediction accuracy (Additional file 1 Figures S1-S3).

This study has several limitations. Although there are explicit diagnostic criteria and reporting management specifications for BD, reporting bias may still exist in the data. Moreover, 1 month may not be an optimal predictive time. We will continue to explore a better predictive time based on the delayed effect of meteorological factors on BD. In addition, our study area is only one municipality directly under the central government in China, which leads to the limitation of extrapolating the conclusion to other regions with different socio-economic and meteorological conditions. In future studies, we will further optimise the settings and perfect the simulation to achieve a better model (Additional file 2: Figures S1–S3).

## Conclusion

1. The average annual incidence of BD in Chongqing from 2009 to 2016 was 29.394/100,000; this level was high, especially in the main urban areas and among the male and pre-educated-child populations.
2. The GA_SVR_MONTH model, which was established based on meteorological factors, showed satisfactory predictive performance. In addition, meteorological factors were proven to obviously improve the accuracies of prediction models of BD incidence in this study. The findings in this study serve as an overview of BD in Chongqing and provide a useful predictive approach for the incidence of infectious disease, which can improve current interventions and public health planning.

## Supplementary information

**Additional file 1: Figure S1–S3** Results about weekly models.

**Additional file 2:** Core code and data set of the establishment of predictive model.

**Additional file 3:** Core code and data set of the establishment of predictive model.

## Data Availability

The data of cases analysed during the study are not publicly available due to regulations, but they can be obtained from the corresponding author on reasonable request. The core code and data set of models in the study are available in the supplemental materials.

## Abbreviations

BD: Bacillary dysentery
GA: Genetic algorithm
MAPE: Mean absolute percent error
MSE: Mean squared error
*R*^2^: Squared correlation coefficient
SVR: Support vector regression model
