# Supplementary material for: Epidemiological characteristics of bacillary dysentery from 2009 to 2016 and its incidence prediction model based on meteorological factors
Source: Environ Health Prev Med. 2019 Dec 28;24:82. doi: 10.1186/s12199-019-0829-1 (PMC6935186; doi:10.1186/s12199-019-0829-1)
Supplement: Supplementary file 1 — Additional file 1: Figure S1–S3 Results about weekly models. [file 12199_2019_829_MOESM1_ESM.dot]

Figure S1. Boruta algorithm relevant feature set screening results for weekly: ten meteorological factors and BD cases are all confirmed important.

**Figure S2.** The comparison of prediction accuracies between GA_SVR_WEEK and SVR_WEEK: the GA_SVR_WEEK in Figure S2 refers to the model that includes the meteorological factors as the independent variable. The SVR_WEEK model in Figure S2 selects only the number of cases as an independent variable. Comparative indices from left to right are the MSE, MAPE and R^2^ of the models. The BD cases and meteorological factors at five time points, which were 3 days, 1 week, 2 weeks, 3 weeks, and 4 weeks prior to the predictive week, were used as independent variables to
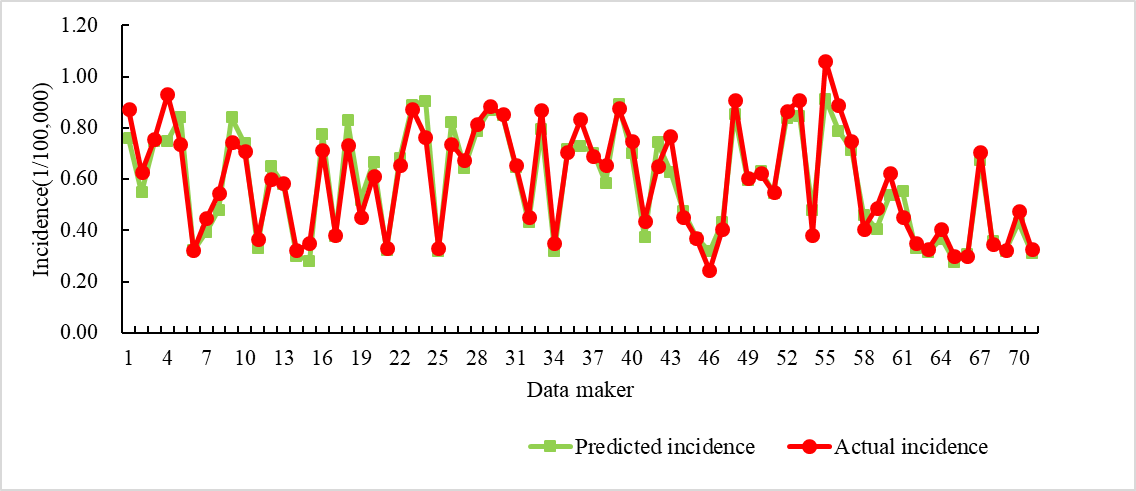
construct separate weekly models.

**Figure S3.** **The incidence predicted by the GA_SVR_WEEK model and the actual incidence: the 10th randomly selected training and test data set.**
